# Genome Sequence of *Geobacillus* sp. Strain ZGt-1, an Antibacterial Peptide-Producing Bacterium from Hot Springs in Jordan

**DOI:** 10.1128/genomeA.00799-15

**Published:** 2015-07-23

**Authors:** Rawana N. Alkhalili, Rajni Hatti-Kaul, Björn Canbäck

**Affiliations:** a Department of Biotechnology, Center for Chemistry and Chemical Engineering, Lund University, Lund, Sweden; b Department of Biology, Microbial Ecology Group, Lund University, Lund, Sweden

## Abstract

This paper reports the draft genome sequence of the firmicute *Geobacillus* sp. strain ZGt-1, an antibacterial peptide producer isolated from the Zara hot spring in Jordan. This study is the first report on genomic data from a thermophilic bacterial strain isolated in Jordan.

## GENOME ANNOUNCEMENT

Lately, the species of the genus *Geobacillus* have been gaining interest as antimicrobial peptide producers (1, 2). *Geobacillus* sp. strain ZGt-1, isolated from the Zara hot spring in Jordan, has been shown to produce an as-yet-uncharacterized antimicrobial peptide (3). In order to screen for the antibacterial protein-encoding genes and to identify potential novel genes associated with antibacterial peptide biosynthesis, we performed a whole-genome sequencing of the bacterium that was already identified by sequencing the PCR-amplified 16S rRNA gene (GenBank accession no. KT026965).

Here, we report the genome sequence of *Geobacillus* sp. strain ZGt-1. Total genomic DNA was extracted from pure cultures of the isolate using ZR Fungal/Bacterial DNA MiniPrep (Zymo Research). A DNA library was constructed using the Nextera protocol with modifications as described earlier (4). Input to the assembly consisted of 680,000 single-end Illumina reads with a length of 151 nucleotides. Quality control was performed by the FastQC version 0.11.2 software (http://www.bioinformatics.bbsrc.ac.uk/projects/fastqc). Reads were assembled using Velveth and Velvetg, both with version 1.2.10 (5). This resulted in an assembly containing 9,625 contigs with a total length of 3.7 million bp. Taking into account that genome sequences from closely related strains were available, it was decided to produce ZGt-1 scaffolds based on the genome sequence of *Geobacillus kaustophilus* HTA426 (GenBank accession number NC_006510.1). This was conducted online with the Scaffold_builder tool (6) using default settings. This resulted in a new assembly with 241 scaffolds and a total length 3,483,107 bp. On this final assembly, gene prediction was carried out with Prodigal version 2_60 using default settings (7). The predicted number of protein-encoding genes was 3,546, which is close to the reported number of genes from *G. kaustophilus* HTA426 (3,397 protein-encoding genes). The GC content was calculated to 52.2% and gene density to 88%.

Genome analysis using antiSMASH version 3.0 software (8) revealed that strain ZGt-1 harbors a lantipeptide biosynthetic gene cluster, where one of the genes encodes for a lantipeptide similar to geobacillin I. The presence of this cluster was also confirmed using BAGEL version 3.0 software (9). The antiSMASH also revealed that the strain harbors another cluster containing a gene encoding for a bacteriocin similar to Linocin M18. A number of putative genes found in the lantipeptide and bacteriocin clusters showed low percentage identity with already described genes. This indicates that the ZGt-1 strain possibly possesses novel genes related to antibacterial peptide production.

Combining the *in silico* analysis of the draft genome of strain ZGt-1 with *in vitro* experimentation is likely to lead to the discovery of novel bioactive compounds.

### Nucleotide sequence accession numbers.

This whole-genome shotgun project has been deposited in DDBJ/EMBL/GenBank under the accession number LDPD00000000. The version described in this paper is the first version, LDPD01000000.
